# Effects of High Al Content on the Phase Constituents and Thermal Properties in NiCoCrAlY Alloys

**DOI:** 10.3390/ma17123025

**Published:** 2024-06-20

**Authors:** Jin Zhang, Zhihua Nie, Chengpeng Tan, Rende Mu, Shilei Li, Xianjin Ning, Chengwen Tan

**Affiliations:** 1 School of Materials Science and Engineering, Beijing Institute of Technology, Beijing 100081, China; 3120211095@bit.edu.cn (J.Z.); nxj@bit.edu.cn (X.N.); tanchengwen@bit.edu.cn (C.T.); 2 Highpure Precision Materials (Suzhou) Co., Ltd., Suzhou 215211, China; tanchengpeng@hppm.net.cn; 3 Aviation Key Laboratory of Science and Technology on Advanced Corrosion and Protection for Aviation Material, AECC Beijing Institute of Aeronautical Materials, Beijing 100095, China; 4 State Key Laboratory for Advanced Metals and Materials, University of Science and Technology Beijing, Beijing 100083, China; lishilei@ustb.edu.cn

**Keywords:** microstructure, phase constituent, synchrotron X-ray diffraction, MCrAlY

## Abstract

MCrAlY (M = Ni and/or Co) metallic coatings are essential for the protection of hot-end components against thermal and corrosion damage. Increasing the Al content is considered a feasible solution to improve the high-temperature performance of MCrAlY coatings. In this paper, the effects of high Al contents (12–20 wt.%) on the phase constituents and cast microstructures in MCrAlY alloys were studied by high-energy X-ray diffraction and electron microscopy techniques combined with phase equilibria calculations. High Al content improved the stability of β, σ, and α phases. Meanwhile, an evolution of the cast microstructure morphology from a dendrite structure to an equiaxed grain structure was observed. The thermal properties were analyzed, which were closely related to the phase constituents and solid-to-solid phase transitions at evaluated temperatures. This work is instructive for developing high-Al-content MCrAlY coatings for next-generation thermal barrier applications.

## 1. Introduction

The hot-end components of modern aero-engines are exposed to complex working conditions during operations, e.g., in sulfur-containing gases at temperatures over 1000 °C [1,2]. To protect the hot-end components, thermal barrier coatings (TBCs) are extensively applied [3]. A standard TBC system consists of a two-layer structure, including an yttria-stabilized zirconia (YSZ) ceramic top coating and a MCrAlY (M = Ni and/or Co) metallic bonding coating [4]. The MCrAlY bond coating consists of an Al-poor γ-Ni solid solution phase and an Al-rich β-(Ni, Co)Al phase, which is designed to protect the substrate from corrosion and oxidation by forming the protective thermally grown oxide (TGO) layer [5,6,7]. The γ-Ni phase serves as the matrix phase in MCrAlY coatings, showing good thermal stability. It provides ductility to the coatings, which can reduce the susceptibility of brittle cracking induced by thermomechanical fatigue [8,9]. The β-(Ni, Co)Al phase is recognized as the oxidation-resistant phase, which provides an Al source to the TGO layer [10]. Typically, MCrAlY coatings are formulated with an appropriate Al content to allow a sufficient β phase to form [11,12].

During high-temperature oxidation, Al is consumed in MCrAlY coatings by the formation of the TGO layer, resulting in an Al depletion zone in the vicinity of the TGO layer. When the Al content decreases to a critical value, other oxides, such as Cr_2_O_3_ and/or Co, Ni, and Cr spinel, can form instead of the more protective Al_2_O_3_, leading to coating failure [13]. Increasing the Al content is considered a potential solution to improve the oxidation resistance of MCrAlY coatings [14,15]. However, increasing the Al content changes the phase constituents and phase transitions in MCrAlY coatings. For instance, increasing the Al content promotes the formation of the minor phases, σ-(Co, Cr) and α-Cr phases [16], which is similar to the effect observed in MCrAlY alloys with the addition of Re [17]. This may potentially deteriorate the mechanical properties of the coatings [18]. Alternatively, it also reduces the coefficient of thermal expansion (CTE) of the coatings and mitigates the thermo-stress at the TGO/coating interface [19]. Consequently, further investigations are essential to understand the effect of Al content on the phase compositions and thermal properties in MCrAlY coatings [20,21].

In this paper, NiCoCrAlY cast model alloys with Al contents of 12%, 16%, and 20% in weight percentage were prepared. The effects of Al content on the phase constituents and phase transitions in NiCoCrAlY alloys were studied by using synchrotron-based X-ray diffraction and electron microscopy techniques combined with calculated phase diagrams. Additionally, the effect of Al content on the CTE was analyzed. Our study provides fundamental results on the effects of high Al content on the phase constituents and thermal properties in NiCoCrAlY alloys, which is instructive for developing high-Al-content MCrAlY coatings for next-generation thermal barrier applications.

## 2. Experiments

### 2.1. Sample Preparations

NiCoCrAlY alloys with nominal compositions of Ni-27.5Co-17Cr-xAl-0.5Y (x = 12, 16, and 20 in weight percentage) were prepared by vacuum induction melting of pure Ni, Co, Cr, Al, and Al-10Y master alloys, which were subsequently cast into a graphite mold. The chemical composition of the as-cast ingots was analyzed by an inductively coupled plasma atomic emission spectrometer (ICP-AES). The composition analysis results are given in Table 1. The NiCoCrAlY alloys were named 12Al, 16Al, and 20Al based on their respective Al contents. The burning loss of Y was observed in 12Al and 20Al alloys. The refining time was cut down in 16Al alloys during vacuum induction melting to reduce the burning loss. The as-cast ingots were cut into blocks with dimensions of 10 mm × 10 mm × 20 mm by spark cutting. The NiCoCrAlY blocks were then heat-treated at different temperatures from 600 to 1300 °C for 2 h, followed by immediate water quenching.

### 2.2. Microstructure Characterizations

The microstructures of 12Al, 16Al, and 20Al alloys were characterized using an OLYMPUS BX51 M optical microscope (OM) (Olympus, Tokyo, Japan), an FEI Quanta 200 (FEI, Hillsboro, OR, USA) FEG environmental scanning electron microscope (SEM) equipped with an energy-dispersive (EDS) detector, and a JEOL JSM-7001F (JEOL, Tokyo, Japan) analytical thermal field-emission SEM equipped with an EDAX Velocity™ electron (EDAX, Mahwah, NJ, USA) backscattering diffraction (EBSD) camera and an EDS detector. EBSD data analysis was carried out using the commercially available TSL-OIM software (Version: 6.2.0 x64). Transmission electron microscopy (TEM) was performed using an FEI Tecnai G2 F20 field-emission system (FEI, Hillsboro, OR, USA) operated at 200 kV.

Synchrotron-based high-energy X-ray diffraction (HE-XRD) experiments were performed in transmission geometry at the beamline BLS12W of the Shanghai Synchrotron Radiation Facility. The photon energy was 100.6 keV (wavelength λ = 0.1236 Å). A PILATUS 1M two-dimensional (2D) detector was used to collect the diffraction patterns with an exposure time of 30 s. Due to the large grains with limited reflections, the samples were continuously rotated around the vertical axis during exposure. The detector’s pixel size was 172 × 172 μm^2^, and the sample-to-detector distance was 0.9 m. The 2D diffraction data were further integrated into one-dimensional (1D) diffraction patterns (intensity versus 2-theta) by the Fit2D software (Version: 12.077).

### 2.3. Phase Equilibria Calculations

Thermodynamic equilibrium calculations were performed using Thermo-Calc^®^ software (Version: 2023.1.111866-468) with the TCNI10^®^ database. The nominal compositions of 12Al, 16Al, and 20Al alloys were used as input data for calculations. The liquid, γ-Ni, and α-Cr phases were described with a substitutional random solution model. The β-(Ni, Co)Al, γ′-Ni_3_Al, σ-(Co, Cr), and Ni_5_Y phases were described with a sublattice model. The Y element mainly exists as the intermetallic compound Ni_5_Y in NiCoCrAlY alloys [22,23]. Here, the Ni_5_Y phase was confirmed by microstructure characterizations. Crystal structures and thermodynamic models of the above-mentioned phases are listed in Table 2 [24,25].

### 2.4. Thermophysical Analysis

Differential scanning calorimetry (DSC) was used to study the phase transitions in the NiCoCrAlY alloys. DSC tests were performed using a Netzsch Model DSC 404F3 (Netzsch, Selb, Germany) differential scanning calorimeter. The testing temperature was 150 to 1250 °C with a heating rate of 10 °C/min. The dimensions of test samples were Φ 3 mm × 1 mm. During the DSC tests, argon was used as the protective gas. The coefficient of thermal expansion was measured by a Netzsch Model DIL 402C (Netzsch, Selb, Germany) dilatometer. The tests were performed in the temperature range from 25 to 1200 °C with a heating rate of 10 °C/min. The dimensions of the test samples were Φ 6 mm × 18 mm. Argon was used as the protective gas in the tests.

## 3. Results

### 3.1. Phase Equilibria Calculations

**Figure 1:**
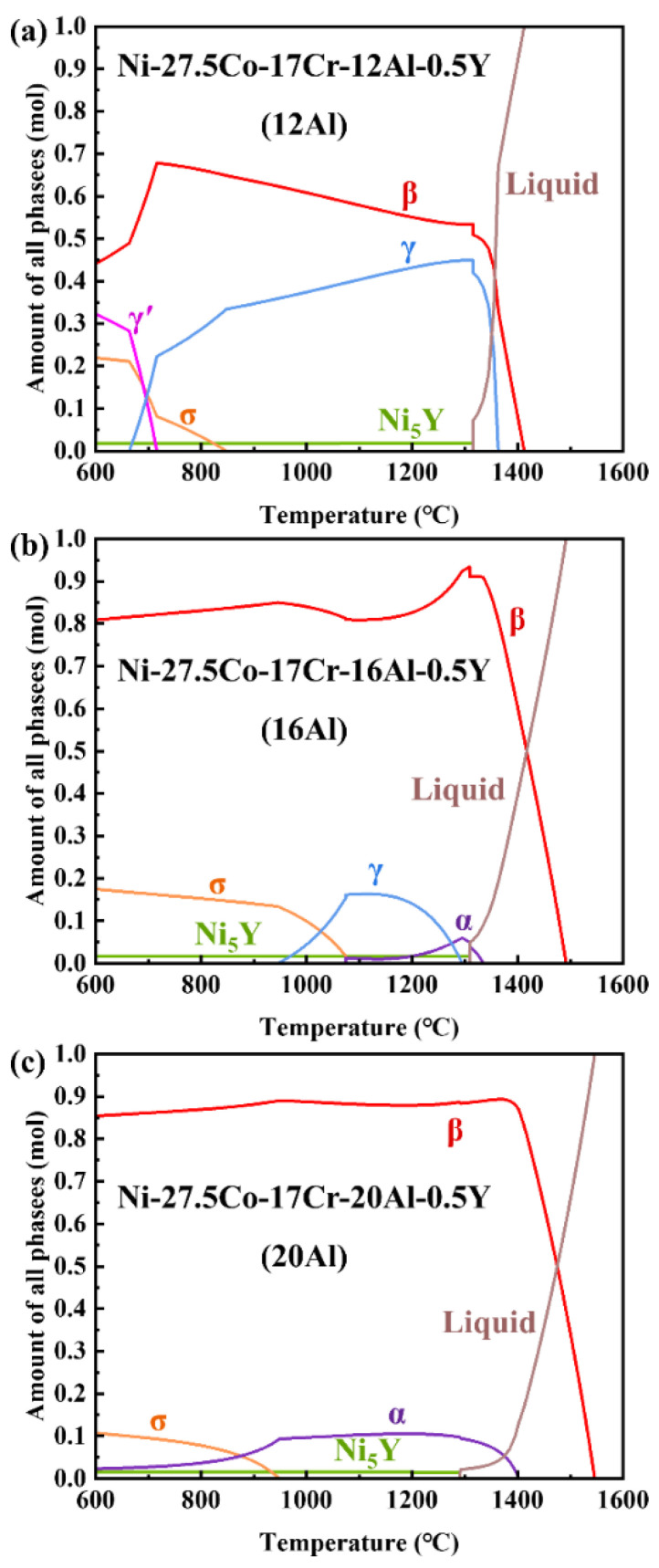
Calculated equilibrium phase fraction versus temperature curves for the Ni-27.5Co-17Cr-xAl-0.5Y (wt.%) alloys supplemented with 12 wt.% Al (**a**), 16 wt.% Al (**b**), and 20 wt.% Al (**c**). The *y*-axis represents the mole fraction of each phase at a given temperature.

Thermo-Calc software uses CALPHAD methodology to predict the thermodynamics and kinetics of multicomponent systems corresponding to real materials. The main equations used in the CALPHAD approach are based on the compound energy formalism, which describes the Gibbs energy of solution phases with sublattices. The thermodynamic modeling in the CALPHAD approach for Ni-based superalloys can be found elsewhere [24]. Figure 1 gives the calculated equilibrium phase fractions as a function of temperature for the 12Al, 16Al, and 20Al alloys. In the 12Al alloy, as shown in Figure 1a, the liquid–solid phase transition (L → β + γ + Ni_5_Y) starts at ~1400 °C and finishes at ~1300 °C. As the temperature decreases below 850 °C, σ and γ′ phases appear through the solid–solid phase transition. It is worth noting that the mole fraction of the Ni_5_Y phase is stable in the whole temperature range below ~1300 °C. When the Al content is increased to 16 wt.%, as shown in Figure 1b, the β phase becomes the primary solid phase in the liquid–solid phase transition (L → β + α + Ni_5_Y). As the temperature decreases, solid–solid phase transitions (α + β → γ + β → σ + β) take place. In the 20Al alloy, as shown in Figure 1c, similar to the 16Al alloy, a liquid–solid phase transition (L → β + α + Ni_5_Y) occurs, in which the β phase is the primary solid phase. When the temperature is decreased below ~950 °C, part of the α phase undergoes an α + β → σ + β transition to precipitate the σ phase. As shown in Figure 1a,b, the addition of Al helps in the formation of the σ phase. However, the fraction of the σ phase decreases with a further increase in Al content to 20 wt.%. This phenomenon may be related to the intense increase in the mole fraction of the α phase in the 20Al alloy.

### 3.2. HE-XRD Results

**Figure 2:**
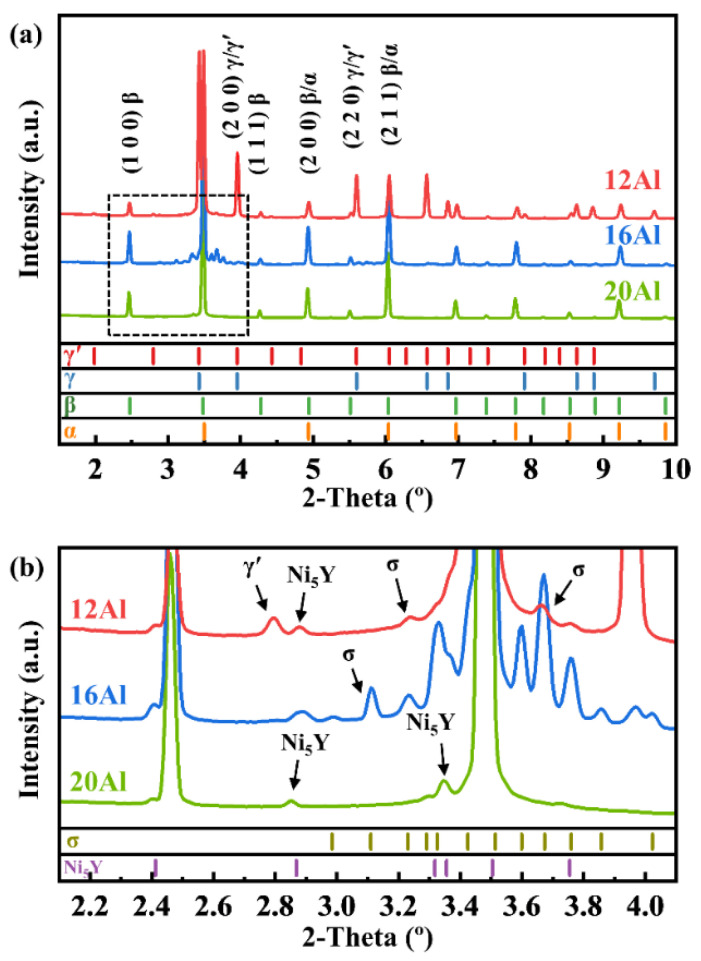
HE-XRD patterns of as-cast 12Al, 16Al, and 20Al alloys. (**b**) is the magnified view of (**a**).

The phase constituents of 12Al, 16Al, and 20Al alloys were studied by the HE-XRD technique. Figure 2 shows the HE-XRD patterns of the as-cast 12Al, 16Al, and 20Al alloys. The areas enclosed by the dashed frame in Figure 2a are enlarged and displayed in Figure 2b. The reflections are indexed by the multiple phases of α, β, γ, γ′, σ, and Ni_5_Y. The crystal structures of the phases are listed in Table 2. The γ phase is a random solid solution and the γ′ phase has the ordered structure of the γ phase [23,24]. The γ′ phase exhibits the fundamental reflections of the γ phase as well as superlattice reflections. The α phase is a body-centered cubic (BCC) random solid solution, while the β phase is an ordered BBC phase. Meanwhile, the α and β phases have similar lattice constants [26]. Therefore, the fundamental reflections of α and β phases are overlapped, except for the superlattice reflections from the β phase. With the assistance of microstructural observations by electron microscope techniques, the phase constituents of as-cast 12Al, 16Al, and 20Al alloys are determined to be β + γ + γ′ + σ + Ni_5_Y, β + σ + Ni_5_Y, and β + α + Ni_5_Y, respectively.

**Figure 3:**
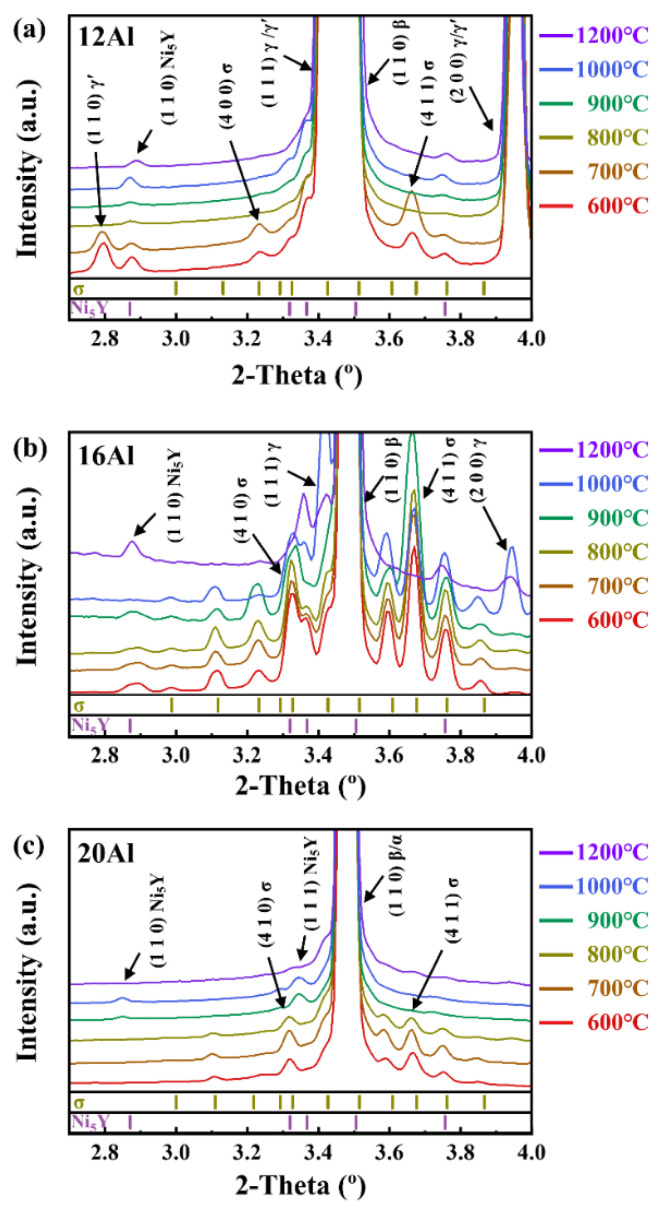
HE-XRD patterns of 12Al (**a**), 16Al (**b**), and 20Al (**c**) alloys quenched at different temperatures.

Figure 3 shows the HE-XRD patterns of the 12Al, 16Al, and 20Al alloys quenched at different temperatures. As shown in Figure 3a, the 12Al alloys quenched at 600 °C and 700 °C contain the same phases as their as-cast state. However, as the quenching temperature is increased to 800 °C, the σ and γ′ phases disappear, indicating that the dissolution temperature of the σ and γ′ phases is between 700 °C and 800 °C. For the 16Al alloys, as shown in Figure 3b, the γ phase appears when the sample is quenched at 1000 °C. This observation is consistent with the calculated equilibrium phase fraction versus temperature curves shown in Figure 1b, in which the γ phase exists in the temperature range between 950 and 1300 °C. When the quenching temperature is increased to 1200 °C, the reflections from the σ phase entirely disappear. As shown in Figure 3c of quenched 20Al alloys, the σ phase is clearly observed for the samples quenched in the temperature range of 600–800 °C. When the quenching temperature is further increased, the reflections of the σ phase disappear. The dissolution temperature of the σ phase is between 800 °C and 900 °C in 20Al alloys. It is noted that the σ phase is not observed in the 20Al alloys in the as-cast state, as shown in Figure 2. The behavior of thermal-induced transition dynamics of the σ phase will be discussed in Section 4.1.

### 3.3. Microstructures of As-Cast Alloys

**Figure 4:**
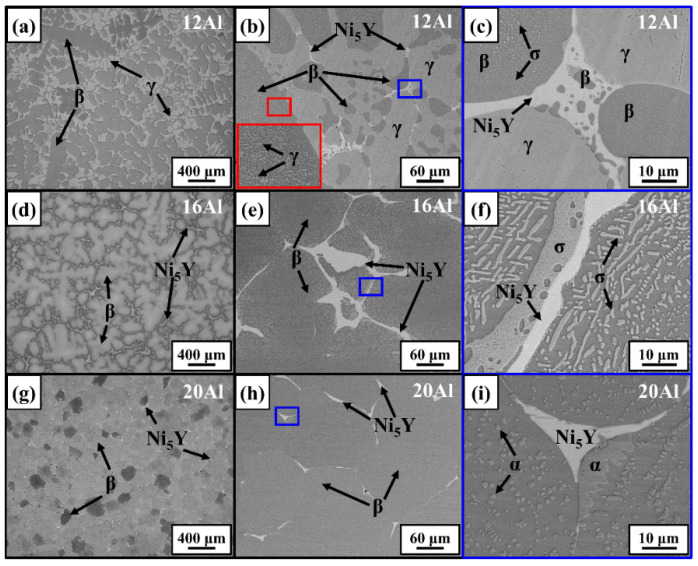
Microstructure of as-cast 12Al (**a**–**c**), 16Al (**d**–**f**), and 20Al (**g**–**i**) alloys. (**a**,**d**,**g**) are OM images at 50×. (**b**,**c**,**e**,**f**,**h**,**i**) are SEM-BSE images. Magnification increases from left to right.

The morphology and distribution of multiple phases in 12Al, 16Al, and 20Al alloys were characterized by optical and electron microscope techniques. Figure 4 gives the OM and backscattered electron (BSE) images of the as-cast alloys. As shown in Figure 4a by OM images, the β phase (dendritic grains in dark contrast) and γ phase (dendritic gap junctions in bright contrast) are observed in as-cast 12Al alloys. The boundary regions between the β and γ phases were further studied by SEM-BSE observations, which are shown in Figure 4b,c. The area enclosed by the red frame in Figure 4b is enlarged and displayed in the insert. As shown in the red frames, a certain amount of γ-phase precipitations (in bright contrast) are observed in the β grains. It is noted that the distribution of γ-phase precipitations in β grains is not uniform. Some β grains do not have γ-phase precipitations. The Ni_5_Y phase is observed at the boundaries between the γ and β phases. The area enclosed by the blue frame in Figure 4b is further enlarged and displayed in Figure 4c. Nano-sized σ precipitations are observed in the β-phase matrix. Some β-phase islands are found in the Ni_5_Y-phase matrix. γ-phase precipitations are not observed in the β grains shown in Figure 4c. Figure 4d–f show the distributions of multiple phases in as-cast 16Al alloys. The 16Al alloys have coarsened β dendrites with Ni_5_Y-phase distributed in the dendritic gap junctions. The σ phase is observed at the edges of the β dendrites, as shown in Figure 4e,f. Figure 4g–i show the distributions of multiple phases in as-cast 20Al alloys. The β-phase equiaxed grains are observed with a grain size of ~200 μm. The Ni_5_Y phase is distributed at the boundaries of β grains. As shown in Figure 4h,i, the sub-micron-sized α phase is evidenced in the β-phase matrix. The multiple phases in as-cast 12Al, 16Al, and 20Al alloys were further verified by chemical composition analysis using the SEM-EDS method. Table 3 lists the chemical compositions of the multiple phases in the as-cast 12Al, 16Al, and 20Al alloys.

**Figure 5:**
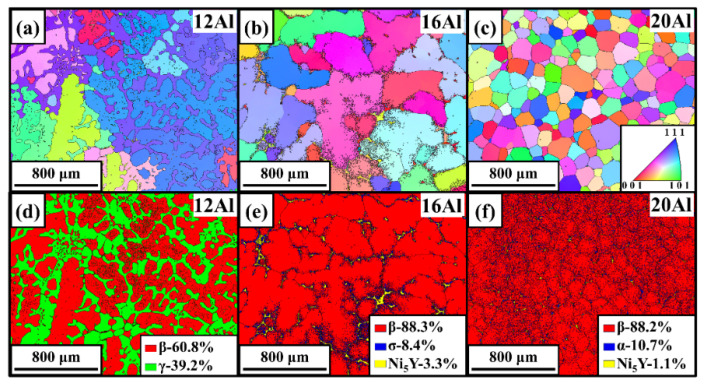
EBSD mappings of as-cast 12Al (**a**,**d**), 16Al (**b**,**e**), and 20Al (**c**,**f**) alloys. (**a**–**c**) are inverse pole figure maps and (**d**–**f**) are phase maps.

The microstructures of multiple phases were quantitatively characterized by the SEM-EBSD technique. The crystal structures listed in Table 2 were used for the indexation of kikuchi patterns. Figure 5 shows the EBSD images for the as-cast 12Al, 16Al, and 20Al alloys. The morphologies of the multiple phases are given by inverse pole figure maps, while the phase contents are revealed by phase maps. Grain boundaries are shown as black lines in Figure 5. Due to the similar crystal structures, the α and β phases are identified with the assistance of EDS analysis. An evolution of as-cast microstructure morphology from dendrite structure to equiaxed grain structure is observed with an increase in Al content from 12 to 20 wt.%. In 12Al alloys, β dendritic grains and γ dendritic gap junctions are observed. Meanwhile, some γ islands are distributed in the β dendritic grains. In as-cast 16Al alloys, the segregation of Ni_5_Y and σ phases at the β grain boundaries is clearly observed. When Al content is increased to 20 wt.%, the Ni_5_Y phase is evidenced at the β grain boundaries and the α phase is distributed in a β matrix. The volume fraction of the β phase is intensively increased when the Al content is increased from 12 to 16 wt.%, which remains invariant as the Al content is further increased to 20%.

**Figure 6:**
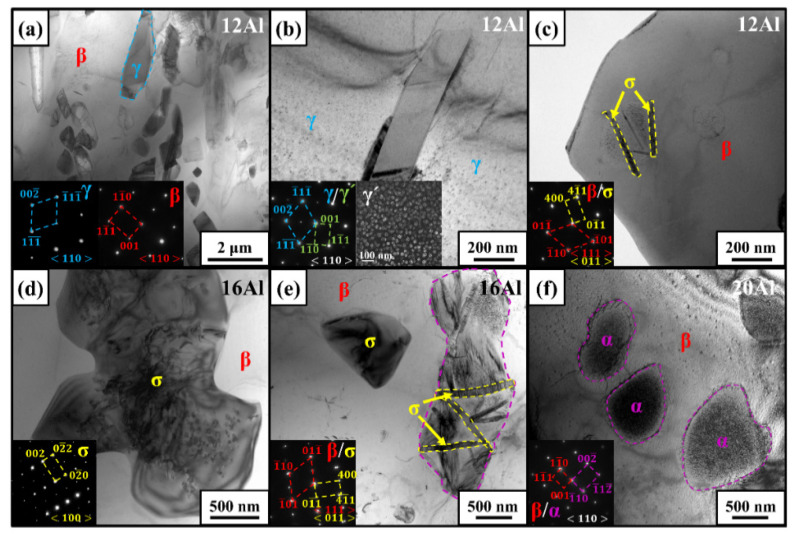
TEM images and the SAED patterns of minor phases in as-cast 12Al (**a**–**c**), 16Al (**d**,**e**), and 20Al (**f**) alloys.

The sub-micron-sized phases in as-cast alloys were characterized by the TEM technique. Figure 6 shows the bright-field images, dark-field images, and the corresponding selected area electron diffraction (SAED) patterns for the as-cast 12Al, 16Al, and 20Al alloys. Figure 6a–c are the TEM images of the as-cast 12Al alloys. As shown in Figure 6a, γ-phase precipitations are observed in the β matrix, where the γ′ phase is not evidenced. However, in γ dendritic gap junctions, as shown in Figure 6b, γ′-phase precipitations are clearly observed, which are uniformly distributed in the γ-phase matrix. Acicular σ-phase precipitates rather than γ-phase precipitations are evidenced in the β matrix shown in Figure 6c. Figure 6d,e are the TEM images of as-cast 16Al alloys. The σ phase is observed in two kinds of morphology. One is a particle implanted in the β matrix and another is acicular precipitated in a matrix circled by dashed purple lines. Here, the matrix circled by dashed purple lines contrasts with the β matrix, which may be the α phase. However, further tests are needed for identification. Figure 6f gives the TEM images of the as-cast 20Al alloys, in which sub-micron-sized α-phase precipitations are observed in the β-phase matrix. The observed α-phase precipitations agree with the SEM observations shown in Figure 4i.

### 3.4. Microstructures of Quenched Alloys

**Figure 7:**
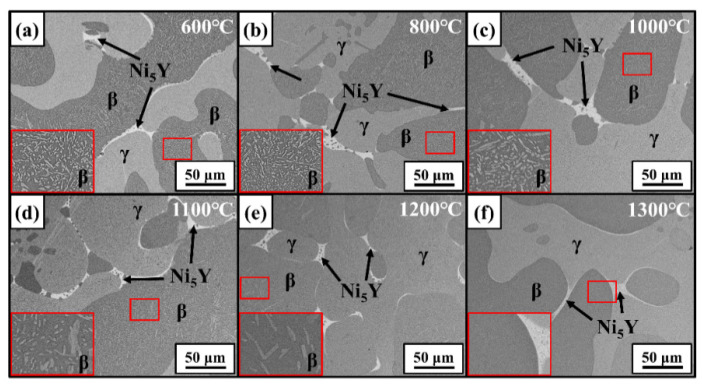
SEM-BSE images of 12Al alloys quenched at 600 °C (**a**), 800 °C (**b**), 1000 °C (**c**), 1100 °C (**d**), 1200 °C (**e**), and 1300 °C (**f**).

**Figure 8:**
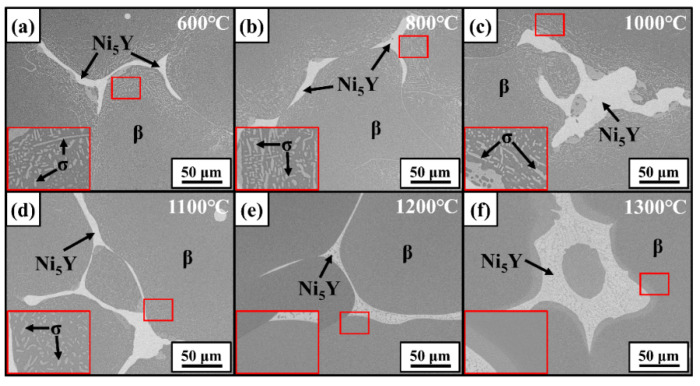
SEM-BSE images of 16Al alloys quenched at 600 °C (**a**), 800 °C (**b**), 1000 °C (**c**), 1100 °C (**d**), 1200 °C (**e**), and 1300 °C (**f**).

**Figure 9:**
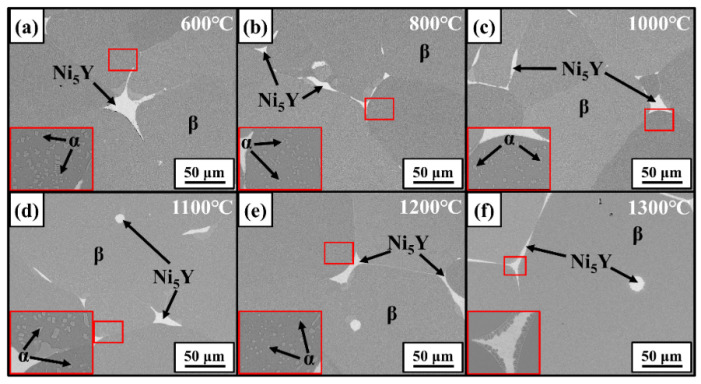
SEM-BSE images of 20Al alloys quenched at 600 °C (**a**), 800 °C (**b**), 1000 °C (**c**), 1100 °C (**d**), 1200 °C (**e**), and 1300 °C (**f**).

Figure 7 gives the SEM-BSE images of the 12Al alloys quenched at different temperatures. The regions enclosed by red frames are enlarged and displayed in the inserts. Similar to the as-cast 12Al alloys, γ, β, and Ni_5_Y phases are observed in the quenched alloys. The γ-phase precipitations are observed in the β dendrites, which dissolve in the β matrix when the quenching temperature is increased to 1300 °C. Figure 8 gives the BSE images of the 16Al alloys quenched at different temperatures. The regions at the β-phase grain boundaries (enclosed by red frames) are enlarged. σ-phase precipitates are evidenced at the β-phase boundaries close to the Ni_5_Y phase, which are dissolved into the β-phase matrix at 1200 °C. Dark contrast appears in the Ni_5_Y phase when the samples are quenched at 1200 °C and 1300 °C, suggesting phase transitions in the Ni_5_Y phase. As shown in Figure 8f, a transition layer is observed at the boundaries between the β and Ni_5_Y phases. As shown in Figure 9, in the quenched 20Al alloys, α-phase precipitations are observed in the β equiaxed grains, which are completely dissolved at 1300 °C. Meanwhile, as shown in the inset of Figure 9f, a transition layer with columnar grains is clearly observed at the boundaries between the β and Ni_5_Y phases.

### 3.5. Thermal Properties

**Figure 10:**
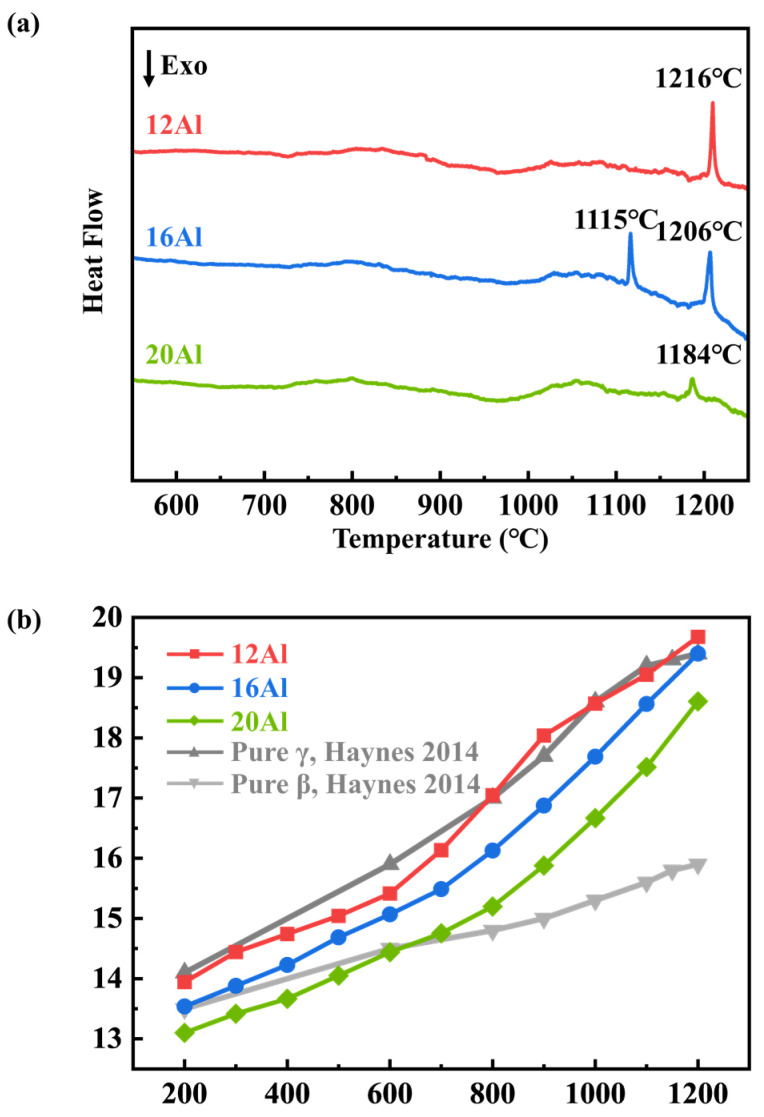
(**a**) DSC curves of 12Al, 16Al, and 20Al alloys. (**b**) Plots of coefficient of thermal expansion for 12Al, 16Al, and 20Al alloys as a function of temperature. The CTE values of the γ and β phases are inserted in (**b**) [27].

The phase transition properties of the as-cast 12Al, 16Al, and 20Al alloys were studied by DSC tests. The DSC curves are presented in Figure 10a. There are endothermic peaks observed at ~1200 °C in all of the alloys. According to the microstructures of quenched alloys, morphology changes are observed in the Ni_5_Y phase or at the boundaries between the β and Ni_5_Y phases when the samples are quenched at 1200 °C and 1300 °C. The endothermic peaks observed at ~1200 °C may be related to the solid–solid phase transitions of the Ni_5_Y phase. Additionally, an endothermic peak appears at 1115 °C in the 16Al alloys, which is attributed to the dissolution of the σ phase into the β matrix. It is noted that the above analysis is based on the microstructure observations of quenched samples in Figure 7, Figure 8 and Figure 9. Further studies are needed to reveal the physical processes of the endothermic reactions. Figure 10b shows the coefficient of thermal expansion (CTE) versus temperature curves for the as-cast 12Al, 16Al, and 20Al alloys, together with CTE values of the γ and β phases [27]. The CTE values in the NiCoCrAlY alloys decrease with the increase in Al content.

## 4. Discussion

### 4.1. Phase Constituents

The thermodynamic parameter, enthalpy of mixing (Δ*H^mix^*), is used here to demarcate solid–solution formation conditions in NiCoCrAlY alloys. In a multi-element alloy system, the constituent elements with a large negative value of Δ*H^mix^* have strong interactions and tend to be neighboring [28]. Elements with a high negative Δ*H^mix^* tend to form an intermetallic compound with stable long-range ordering [29]. The values of Δ*H^mix^* between elements in the NiCoCrAlY system are listed in Table 4 [30], which are used to discuss the phase constituents in the alloys. Y has a high negative value of Δ*H^mix^* with Ni, Co, and Al. Therefore, a stable intermetallic compound Ni_5_Y phase is formed in the NiCoCrAlY alloys, which is extensively evidenced at the grain boundaries by SEM-BSE observations. The chemical composition of the Ni_5_Y phase is further examined by the EDS analysis (see Table 3), showing a high content of Ni, Co, and Al elements in the compound.

Al element has a large negative value of Δ*H^mix^* with Ni and Co. Al tends to destabilize the solid solution, which promotes the formation of the β-(Ni, Co)Al phase [31]. As shown in the phase equilibria calculations and SEM-BSE observations, the β phase is the primary solidification phase in the NiCoCrAlY alloys. When the Al content is increased from 12 to 20 wt.%, the volume fraction of the β phase is intensively increased. Meanwhile, the fraction of the γ phase becomes less with the increase in Al content, which vanishes in 20Al alloys. The β phase is normally recognized as the Al reservoir [15]. The addition of Al promotes the formation of an Al-rich β-(Ni, Co)Al phase instead of an Al-poor γ-Ni phase in the alloys. Due to the limited solubility of Cr in the β phase [32], the Cr element presents mainly in the σ and/or α phases in the 16Al and 20Al alloys. The formation of the α phase is promoted in the NiCoCrAlY alloys as the Al content increases (see Figure 1, Figure 2, and Figure 4). As evidenced by HE-XRD and SEM observations in Figure 2, Figure 4, and Figure 5, the σ phase is not observed in the as-cast 20Al alloys. This comes into conflict with the phase equilibria calculations in Figure 1. The α-to-σ phase transformation is not accomplished in the 20Al alloys during the casting process, which is attributed to the slow kinetics [33]. However, the σ phase reappears when the samples are further heat-treated, as shown in Figure 3. A similar phenomenon is reported in high-Cr ferritic steels [33].

### 4.2. Solidification Behaviors

**Figure 11:**
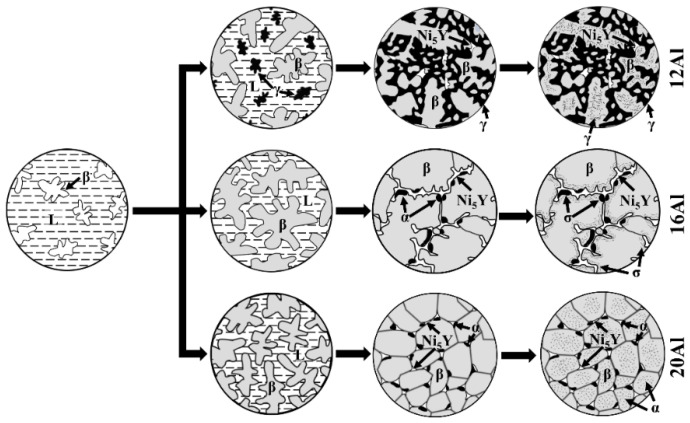
Schematic diagrams of the liquid-to-solid and solid-to-solid phase transitions in the casting process for 12Al, 16Al and 20Al alloys.

The experimental results and phase equilibria calculations suggest that the increase in Al content in the NiCoCrAlY alloys not only changes the phase constituents but also widens the solidification temperature ranges. As a result, the as-cast microstructures are significantly affected by the Al content. Schematic diagrams of the liquid-to-solid and solid-to-solid phase transitions in the casting process were proposed for the NiCoCrAlY alloys and are shown in Figure 11. In the 12Al alloys, β dendrites are solidified in the liquid, followed by the co-solidification of γ and β phases in β dendritic gap junctions, forming a typical eutectic solidification microstructure [34]. The Ni_5_Y phase is generated at the end of solidification processing. When the 12Al alloys are further cooled down, the γ phase is precipitated in some β grains. The 16Al alloys exhibit coarsened β dendrites, which is attributed to the wide solidification temperature range. Subsequently, the α and Ni_5_Y phases are solidified at the edges of the β dendrites. Solid-phase transitions of α + β → γ + β → σ + β occur when the 16Al alloys are further cooled down to room temperature. During the solidification of the 20Al alloy, a mushy zone is formed due to the extensive solidification temperature range [35]. Due to the limited chemical segregation in solid–liquid components, numerous β-crystal nuclei are formed in the mushy zone, which grow and develop into equiaxed β grains during solidification. Ultimately, the α and Ni_5_Y phases are solidified at the boundaries between the β equiaxed grains. When the 20Al alloys are further cooled down, α precipitations are formed in the β grains. It should be noted that the cooling rate of the thermal spraying process is much faster than that of the casting process in the vacuum induction melting [17,18]. A significant difference in microstructure can be predicted. On one hand, a high cooling rate can refine the grains and improve the uniformity of chemical composition. On the other hand, it may prevent some phases (i.e., σ and α phases) from precipitating during the cooling process [18]. Further studies are needed to reveal the effects of high Al content on the microstructural evolutions in NiCoCrAlY coatings prepared by the thermal spraying process.

### 4.3. Thermal Expansion Behaviors

The thermal expansion behaviors of NiCoCrAlY alloys are closely related to the phase constituents. As shown in Figure 10b, the CTE value of the γ phase is higher than that of the β phase [27]. Moreover, the CTE of the β phase is higher than that of the α phase [36,37]. When the Al content is increased from 12 to 16 wt.%, the decrease in CTE is attributed to the increase in the β-phase fraction. In addition, there is about a 10% volume fraction of α phase exiting in the 20Al alloys, which is stable up to 1200 °C. Therefore, the CTE of the 20Al alloys is lower than that of the 16Al alloys. As shown in Figure 10b, there is not a linear relationship between CTE and temperature. The CTE of the 12Al alloys increases intensively from 600 to 900 °C, which can be attributed to the γ′ + σ → γ + β phase transition [27]. In 16Al and 20Al alloys, there is a slope change in CTE at 700–800 °C, which is related to the ductile–brittle transition of the β phase [38]. There are several phases existing in NiCoCrAlY alloys, and each phase has different thermal properties. Fractional calculations provide an effective tool to evaluate the thermal properties in advanced materials with multiple phases [39,40]. Further studies including experiments and first-principles calculations are needed to quantitatively evaluate the thermal properties in bulk NiCoCrAlY samples.

## 5. Conclusions

The effects of high Al content on the phase constituents and solidification behaviors of NiCoCrAlY alloys were investigated by using HE-XRD and electron microscopy techniques combined with phase equilibria calculations. The relations between phase constituents and thermal properties were discussed. The following conclusions can be drawn:

1. The phase constituents of as-cast 12Al, 16Al, and 20Al alloys are determined to be β + γ + γ′ + σ + Ni_5_Y, β + σ + Ni_5_Y, and β + α + Ni_5_Y, respectively, which are verified by the HE-XRD and electron microscopy analyses with the assistance of thermodynamic equilibrium calculations. The β phase is the primary solid phase in NiCoCrAlY alloys. High Al content improves the stability of the β, σ, and α phases.
2. The solidification temperature ranges are widened with an increase in Al content. Thus, an evolution of the as-cast microstructure morphology from a dendrite structure to an equiaxed grain structure is observed. Based on the experimental results and phase equilibria calculations, schematic diagrams of the liquid-to-solid and solid-to-solid phase transitions in the casting process are illustrated.
3. The coefficient of thermal expansion is analyzed. It is closely related to the phase constituents and solid-to-solid phase transitions at elevated temperatures.

## Figures and Tables

**Table 1:** Chemical compositions of 12Al, 16Al, and 20Al alloys by ICP-AES (wt.%).

	Ni	Co	Cr	Al	Y
12Al	Bal.	27.5	16.84	11.46	0.27
16Al	Bal.	27.28	16.99	16.55	0.58
20Al	Bal.	27.46	17.58	21.22	0.24

**Table 2:** Summary of crystal structures and thermodynamic models used in phase equilibria calculations [24,25].

Phase Name	Strukturbericht Symbol	Pearson Symbol Prototype Space Group	Thermodynamic Model
Liquid	-	-	(Ni, Co, Cr, Al, Y)_1_
γ-Ni	A1	cF4–Cu $Fm\bar{3}m$	(Ni, Co, Cr, Al, Y)_1_(VA)_1_
α-Cr	A2	cI2–W $Im\bar{3}m$	(Ni, Co, Cr, Al, Y, VA)_1_(VA)_3_
γ′-Ni_3_Al	L12	cP4–Cu_3_Au $Pm\bar{3}m$	(Ni, Co, Cr, Al, Y)_0.75_(Ni, Co, Cr, Al, Y)_0.25_(VA)_1_
β-(Ni, Co)Al	B2	cP2–CsCl $Pm\bar{3}m$	(Ni, Co, Cr, Al, Y, VA)_0.5_(Ni, Co, Cr, Al, Y, VA)_0.5_(VA)_3_
σ-(Co, Cr)	D8b	tP30–CrFe $P4\bar{2}/mnm$	(Ni, Co, Cr, Al)_10_(Ni, Co, Cr, Al)_4_(Ni, Co, Cr, Al)_16_
Ni_5_Y	D2d	hP6–CaCu_5_ P6/mmm	(Ni, Al)_5_(Y)_1_

Note: the term “VA” denotes the vacancies in the thermodynamic model.

**Table 3:** Chemical compositions of main phases in the as-cast 12Al, 16Al, and 20Al alloys by EDS (wt.%). Values are presented as an average of three tests.

		Ni	Co	Cr	Al	Y
12Al	β	47.8 ± 1.1	25.0 ± 1.0	11.5 ± 1.0	15.7 ± 0.8	-
	γ	37.6 ± 0.2	33.9 ± 0.3	22.5 ± 0.4	6.0 ± 0.2	-
	Ni_5_Y	51.7 ± 1.1	19.2 ± 0.4	5.3 ± 0.3	5.8 ± 0.5	17.9 ± 1.1
16Al	β	41.4 ± 0.7	27.6 ± 0.4	13.2 ± 1.0	17.7 ± 1.0	-
	σ	12.7 ± 0.4	34.9 ± 0.3	49.9 ± 0.3	2.6 ± 0.2	-
	Ni_5_Y	42.1 ± 0.7	25.1 ± 0.4	8.1 ± 0.3	6.6 ± 0.2	18.1 ± 0.6
20Al	β	35.2 ± 0.4	29.9 ± 1.0	13.3 ± 1.9	21.5 ± 1.2	-
	α	5.7 ± 0.9	22.9 ± 0.3	69.3 ± 1.1	2.1 ± 0.5	-
	Ni_5_Y	41.0 ± 0.4	21.7 ± 0.7	5.9 ± 0.8	8.1 ± 0.2	23.4 ± 0.3

**Table 4:** The values of Δ*H^mix^* by Miedema’s model (unit: kJ/mol) [28].

	Ni	Co	Cr	Al	Y
Ni	-	0	−7	−22	−31
Co	0	-	−4	−19	−22
Cr	−7	−4	-	−10	11
Al	−22	−19	−10	-	−38
Y	−31	−22	11	−38	-

## Data Availability

The data that support the findings of this study are available from the corresponding author upon reasonable request.
